# Association of Frailty and Its Trajectories With the Risk of Cardiovascular–Kidney–Metabolic Syndrome Progression: A Longitudinal Cohort Study

**DOI:** 10.1111/ggi.70627

**Published:** 2026-07-02

**Authors:** Haoyu Yang, Zhongli Wang, Wuyong Yi, Ling Men, Riming He, Shudong Yang

**Affiliations:** ^1^ Department of Nephrology Shenzhen Traditional Chinese Medicine Hospital, the Fourth Clinical Medical College of Guangzhou University of Chinese Medicine Shenzhen China; ^2^ Medical College of Acu‐Moxi and Rehabilitation Guangzhou University of Chinese Medicine Guangzhou China

**Keywords:** cardiovascular disease, cardiovascular‐kidney‐metabolic syndrome, epidemiology, frailty, trajectory

## Abstract

**Aims:**

Frailty reflects a decline in physiological reserve and has been widely recognized as an important risk factor for cardiovascular disease (CVD). However, the relationships between frailty and the progression of the cardiovascular–kidney–metabolic (CKM) syndrome, as well as between dynamic frailty trajectories and CVD risk, have not been systematically investigated.

**Methods:**

A total of 5624 participants from the China Health and Retirement Longitudinal Study (CHARLS) were included. The association between baseline frailty and CVD risk was analyzed using the Cox proportional hazards model and the restricted cubic spline analyses. We used Group‐based trajectory modeling (GBTM) to identify trajectories of frailty development during the follow‐up periods from 2011 to 2018.

**Results:**

Higher baseline frailty index (FI) was significantly associated with CVD risk in individuals with CKM syndrome stages 0–3; each 0.1 increase in FI was associated with a 33% higher risk of CVD after full adjustment (HR 1.33, 95% CI 1.26–1.40). Cumulative frailty burden, assessed using the cumulative FI (cumFI), showed a strong graded association with CVD risk. Three distinct frailty trajectories were identified. Compared with the low‐level increasing trajectory, moderate‐level and high‐level increasing trajectories were associated with higher CVD risk (HR = 4.63, 95% CI: 3.50–6.14, HR = 9.33, 95% CI: 6.89–12.64). These associations were consistent across multiple sensitivity analyses.

**Conclusions:**

Frailty is associated with the risk of CKM syndrome progression. Beyond baseline assessment, cumFI and frailty trajectories provide prognostic information for cardiovascular risk. Dynamic assessment and early intervention targeting frailty may offer a strategy for preventing CVD.

## Graphical abstract

In a longitudinal cohort of individuals with early‐stage cardiovascular–kidney–metabolic (CKM) syndrome, higher baseline frailty, greater cumulative frailty burden, and unfavorable frailty trajectories were independently associated with an increased risk of incident cardiovascular disease (CVD), highlighting frailty as a dynamic marker for early cardiovascular risk stratification.

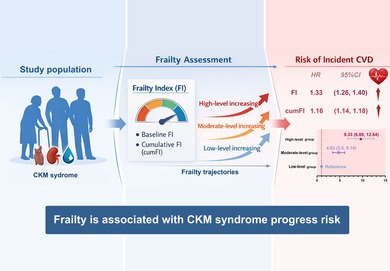

## 1 Introduction

With the rapid acceleration of global population aging, cardiovascular disease (CVD) remains the leading contributor to disease burden and mortality among older adults worldwide [1]. To more comprehensively capture the interconnected nature of cardiometabolic disorders, the American Heart Association recently proposed the concept of cardiovascular–kidney–metabolic (CKM) syndrome, which integrates CVD, chronic kidney disease, and metabolic dysfunction into a unified clinical framework [2, 3]. CKM syndrome is classified into stages 0 through 4 according to disease progression, underscoring the importance of early identification and refined risk stratification before the onset of overt cardiovascular events. The pathophysiology of CKM syndrome is multifactorial, involving insulin resistance, oxidative stress, chronic low‐grade inflammation, and lipotoxicity [4], which collectively contribute to progressive multisystem dysfunction. Clinically, CKM syndrome is strongly associated with an elevated risk of cardiovascular events and renal failure, highlighting its substantial relevance for both clinical management and public health prevention strategies.

Frailty is an age‐related clinical syndrome characterized by a decline in physiological reserve and an increased vulnerability to stressors, which in turn predisposes individuals to adverse health outcomes [5]. Beyond merely coexisting with multiple chronic conditions, frailty is closely linked to disease severity and progression, particularly among patients with CVD and chronic kidney disease [6, 7], where it substantially influences therapeutic decision‐making and long‐term prognosis. Emerging evidence further suggests that frailty is strongly aligned with CKM syndrome staging, with frail individuals experiencing markedly elevated risks of cardiovascular events and all‐cause mortality. Collectively, these findings position frailty as a critical yet underrecognized phenotype within the CKM syndrome continuum [8].

Although prior studies have demonstrated that frailty is associated with an elevated risk of CVD [7, 9], most investigations have been conducted in general populations and have not specifically focused on individuals in CKM syndrome stages 0–3. This subgroup represents a critical phase of cardiometabolic dysfunction before clinical CVD and constitutes an important window for refined risk stratification and early preventive intervention. Furthermore, several previous studies [7, 10, 11] have relied on cross‐sectional designs or relatively short follow‐up periods and have conceptualized frailty as a static condition rather than a dynamic continuum. In these analyses, frailty was typically assessed at a single time point or characterized by limited changes over one follow‐up interval, which may be insufficient to capture the long‐term evolution of frailty and its potentially complex relationship with cardiovascular risk. Consequently, robust longitudinal evidence elucidating the association between frailty trajectories and incident CVD remains scarce.

Against this background, the present study leveraged data from the China Health and Retirement Longitudinal Study (CHARLS), a nationally representative prospective cohort, to systematically investigate the associations of baseline frailty status, cumulative frailty burden, and longitudinal frailty trajectories with the risk of incident CVD. By integrating both static and dynamic dimensions of frailty assessment, this study aims to advance the understanding of how frailty is associated with cardiovascular risk across CKM syndrome stages 0–3. These findings may inform earlier identification of high‐risk individuals and support the development of more precise, stage‐specific cardiovascular prevention strategies in middle‐aged and older adults.

## 2 Methods

### 2.1 Study Population

Data for this study were derived from the CHARLS, a nationally representative, large‐scale prospective cohort study conducted by the National School of Development at Peking University. CHARLS was designed to collect high‐quality microdata on Chinese households and individuals aged 45 years and older, using a multistage, stratified probability sampling strategy with probability proportional to size [12]. The survey covers 150 counties or districts across 28 provinces, autonomous regions, and municipalities in China. The baseline survey was conducted in 2011, with follow‐up assessments administered biennially. To date, five waves of data have been publicly released, including those from 2011 (baseline), 2013, 2015, 2018, and 2020. This study was conducted in accordance with the principles of the Declaration of Helsinki. The CHARLS protocol was approved by the Biomedical Ethics Review Committee of Peking University (IRB No. IRB00001052‐11015). All participants provided written informed consent prior to enrollment. Field staff received standardized and systematic training, and data were collected through face‐to‐face interviews using structured questionnaires. Detailed information regarding the CHARLS study design and data access procedures is available on the official website (http://charls.pku.edu.cn/en).

**FIGURE 1:**
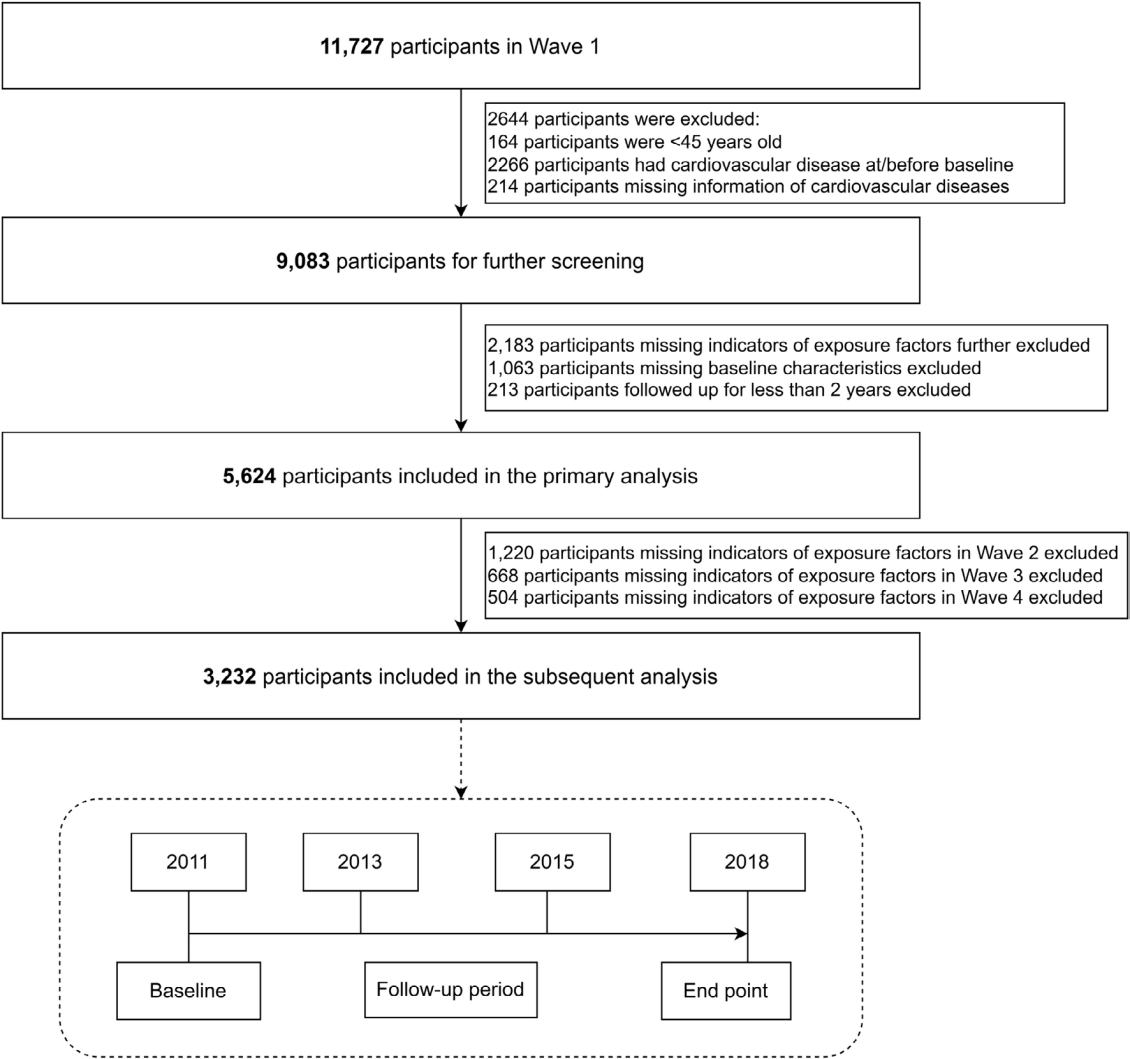
Selection process of the study population.

The participant selection process is illustrated in Figure 1. Briefly, individuals with prevalent CVD at baseline (*n* = 2266), missing CVD information (*n* = 214), or aged younger than 45 years (*n* = 164) were initially excluded. We further excluded participants with missing data required for the calculation of the baseline frailty index (FI) (*n* = 2183), missing baseline covariates or indicators necessary for CKM syndrome staging (*n* = 1063), and those with a follow‐up duration of less than 2 years (*n* = 213). After applying these inclusion and exclusion criteria, a total of 5624 participants were eligible for the primary analyses. For the frailty trajectory analysis, an additional 2392 participants were excluded based on similar criteria, yielding a subset with sufficient longitudinal FI measurements for trajectory modeling.

### 2.2 Data Assessment

#### 2.2.1 Outcome Ascertainment

The primary outcome of interest was the incidence of CVD, including cardiac events and stroke, during a 7‐year follow‐up period. Follow‐up time was calculated from the date of study enrollment to the occurrence of the first CVD event or the date of the last available contact, whichever came first. Incident CVD events were ascertained using two standardized, self‐reported questionnaire items: “Have you ever been diagnosed by a physician with heart attack, coronary heart disease, angina pectoris, congestive heart failure, or other heart‐related conditions?” and “Have you ever been diagnosed by a physician with stroke?” Participants who responded “yes” to either question were classified as having incident CVD during follow‐up [7, 13].

#### 2.2.2 Assessment of Frailty

Frailty was quantified using the FI, a well‐established measure based on the cumulative burden of age‐related health deficits. In this study, the FI was constructed in accordance with previously described standard procedures [14, 15]. Using data from the CHARLS, a total of 30 variables were selected to construct the FI, encompassing multiple domains of health, including chronic diseases (excluding heart disease and stroke), symptoms, disabilities, physical functioning, depressive symptoms, and cognitive performance (see Table S1). Variables 1–29 were dichotomized according to predefined cut‐off values and coded as 1 (deficit present) or 0 (deficit absent). The 30th variable, cognitive score, was treated as a continuous measure ranging from 0 to 1, with higher values indicating poorer cognitive function. For each participant, the FI was calculated by summing the number of deficits present and dividing by the total number of items (30). Accordingly, the FI ranged from 0 to 1, with higher values reflecting greater frailty severity. Consistent with previous studies, participants were classified into three frailty categories: robust (FI ≤ 0.10), pre‐frail (0.10 < FI < 0.25), and frail (FI ≥ 0.25). Frailty trajectories were assessed using frailty status at baseline and across three subsequent follow‐up waves. In addition, the cumulative frailty index (cumFI) was calculated as the sum of FI values at baseline and the three follow‐up assessments, representing the long‐term cumulative burden of frailty over time.

#### 2.2.3 Definition of CKM Syndrome Stages

According to the American Heart Association (AHA) Presidential Advisory [2], CKM syndrome represents an integrated framework that captures the interrelated pathophysiology of CVD, chronic kidney disease, and metabolic disorders. The CKM syndrome is categorized into five progressive stages (0–4) based on the presence and severity of metabolic risk factors, kidney dysfunction, and cardiovascular involvement. Briefly, stage 0 refers to individuals without identifiable CKM syndrome risk factors; stage 1 includes individuals with excess adiposity, central obesity, or adipose tissue dysfunction in the absence of chronic kidney disease (CKD); stage 2 comprises individuals with established metabolic risk factors—such as hypertriglyceridemia, hypertension, metabolic syndrome, or type 2 diabetes—or those with moderate‐ to high‐risk CKD, or both; stage 3 is defined by the presence of high‐risk CKD or subclinical CVD; stage 4 represents individuals with clinically manifest CVD [16]. Subclinical CVD was identified based on the Framingham risk score and the presence of very high‐risk CKD, in accordance with prior definitions [17]. The Framingham risk score integrates major traditional cardiovascular risk factors, including age, sex, blood pressure, smoking status, and diabetes. CKD stages were classified according to the Kidney Disease: Improving Global Outcomes (KDIGO) guidelines [18]. Detailed criteria for CKM syndrome staging are provided in Tables S2 and S3.

#### 2.2.4 Data Collection

The baseline survey collected demographic characteristics, including age, sex, place of residence, educational attainment, and marital status. Anthropometric measurements, including height, weight, waist circumference, systolic blood pressure (SBP), and diastolic blood pressure (DBP), were obtained by trained staff following standardized protocols. Information on health‐related conditions and behaviors was collected using structured questionnaires and included smoking status, alcohol consumption, sleep status, and self‐reported physician‐diagnosed comorbidities, such as hypertension, diabetes mellitus, pulmonary disease, cancer, and kidney disease. As part of the laboratory assessment, fasting blood samples were collected to measure biochemical parameters, including fasting blood glucose (FBG), glycated hemoglobin (HbA1c), triglycerides (TG), total cholesterol (TC), high‐density lipoprotein cholesterol (HDL‐C), low‐density lipoprotein cholesterol (LDL‐C), serum creatinine (Scr), blood urea nitrogen (BUN), uric acid (UA), and C‐reactive protein (CRP). Multiple imputation was applied to address missing values in the study variables (Figure S1).

#### 2.2.5 Statistical Analysis

Baseline characteristics of the study population were summarized according to frailty status. Continuous variables were presented as mean ± standard deviation (SD) or median with interquartile range (IQR), as appropriate based on their distribution. Between‐group differences were assessed using one‐way analysis of variance (ANOVA) for normally distributed continuous variables and the Kruskal–Wallis *H* test for non‐normally distributed variables. Categorical variables were expressed as counts (percentages) and compared using the *χ* ^2^ test or Fisher's exact test, as appropriate.

Cumulative incidence of CVD across different frailty status groups was illustrated using Kaplan–Meier curves, and between‐group differences were compared using the log‐rank test. Cox proportional hazards regression models were applied to examine the associations between the baseline FI and the risks of incident CVD, with hazard ratios (HR) and corresponding 95% confidence intervals (95% CI) estimated. An unadjusted model (Model 1) and three progressively adjusted models were constructed. Model 2 was adjusted for age and sex. Model 3 was further adjusted for marital status, educational attainment, smoking status, alcohol consumption, and body mass index (BMI). Model 4 additionally adjusted for laboratory biomarkers, including HbA1c, Scr, BUN, UA, LDL‐C, and TG. The proportional hazards assumption was assessed using Schoenfeld residuals. Using the same Cox proportional hazards regression models, the association between cumFI and the risks of incident CVD was further evaluated. To explore potential non‐linear associations between the FI and CVD risk, restricted cubic spline (RCS) regression models were employed.

Group‐based trajectory modeling (GBTM) was applied to identify latent developmental patterns of the FI across follow‐up periods. Using FI measurements obtained in 2011, 2013, 2015, and 2018, trajectories were modeled among participants who completed all four survey waves. A censored normal distribution was specified to accommodate the continuous nature of the FI. Models with one to five trajectory groups were sequentially fitted. The optimal number of trajectories was determined based on model fit indices, including the Akaike Information Criterion (AIC) and Bayesian Information Criterion (BIC), the average posterior probability of assignment (AvePP), and the proportion of participants in each trajectory group. Although AIC and BIC continued to decrease as the number of groups increased, the three‐group model was selected as optimal based on classification accuracy, adequate group size, and clinical interpretability. The proportions of participants in the three groups were 34.50%, 47.00%, and 18.50%, with corresponding AvePP values of 0.93, 0.92, and 0.95. Detailed model‐fitting results are shown in Table S4. The identified frailty trajectories were labeled as low‐level increasing, moderate‐level increasing, and high‐level increasing trajectories. Cox proportional hazards regression models were subsequently used to evaluate the associations between frailty trajectory membership and CVD risk, with sequential adjustment for potential confounders.

All statistical analyses were performed using R software (version 4.4.3). A two‐sided *p* < 0.05 was considered statistically significant.

### 2.3 Sensitivity Analyses

To assess the robustness of the primary findings, a series of sensitivity analyses were conducted. First, the main analyses were repeated after excluding outliers in baseline FI to evaluate the potential influence of extreme frailty values on the observed associations. Second, logistic regression models were applied to examine the association between FI and CVD risk, thereby assessing the consistency of results across different statistical modeling approaches. Third, to address potential overlap between FI components and CKM syndrome stage variables, additional analyses were performed using a modified FI that excluded hypertension and diabetes. Fourth, to further assess the potential influence of differential healthcare contact on outcome detection, the baseline FI model was additionally adjusted for baseline healthcare utilization. Fifth, to further reduce the possibility that baseline frailty reflected elevated underlying cardiovascular risk, an additional sensitivity analysis was conducted with further adjustment for baseline Framingham risk score. In addition, to minimize the possibility of reverse causation, where baseline frailty might reflect subclinical or undiagnosed cardiovascular conditions, participants who developed CVD by the second follow‐up wave were excluded, and the primary analyses were re‐performed in the remaining cohort.

## 3 Results

### 3.1 Baseline Characteristics of the Study Population

A total of 5624 participants were included in the present analysis and were classified into three groups according to baseline frailty status: non‐frail (*n* = 2746), pre‐frail (*n* = 2325), and frail (*n* = 553). Baseline characteristics of the study population stratified by frailty status are summarized in Table 1. Compared with non‐frail participants, those in the pre‐frail and frail groups were older, with a progressively higher proportion of individuals aged ≥ 60 years. The proportion of women increased significantly with worsening frailty status, as did the proportion of participants residing in rural areas. Participants in the frail group were more likely to have lower educational attainment. Regarding lifestyle factors, the frail group exhibited lower proportions of current smoking and alcohol consumption, but higher proportions of former smoking and former drinking compared with the other two groups. The prevalence of poor sleep quality increased markedly across increasing frailty categories. In parallel, SBP levels showed a graded increase with greater frailty severity. In terms of clinical and biochemical profiles, frail participants demonstrated higher levels of systemic inflammation and metabolic dysregulation, as reflected by elevated concentrations of CRP, FBG, HbA1c, TC, and TG. Moreover, the distribution of CKM syndrome stages differed significantly across frailty groups.

**TABLE 1:** Baseline characteristics of CKM syndrome stage 0–3 participants according to frailty status.

	Robust (*N* = 2746)	Pre‐frail (*N* = 2325)	Frail (*N* = 553)	*p*
Age				
Mean (SD)	56.37 (8.268)	58.35 (8.351)	62.28 (8.369)	< 0.001
Median [Min, Max]	56.00 [45.00, 101.0]	58.00 [45.00, 90.00]	62.00 [45.00, 96.00]	
Age_group				
< 60	1883 (68.6%)	1346 (57.9%)	216 (39.1%)	< 0.001
≥ 60	863 (31.4%)	979 (42.1%)	337 (60.9%)	
Sex				
Female	1221 (44.5%)	1310 (56.3%)	349 (63.1%)	< 0.001
Male	1525 (55.5%)	1015 (43.7%)	204 (36.9%)	
Residence				
Urban	1074 (39.1%)	778 (33.5%)	135 (24.4%)	< 0.001
Rural	1672 (60.9%)	1547 (66.5%)	418 (75.6%)	
Education				
Middle school or below	2335 (85.0%)	2119 (91.1%)	539 (97.5%)	< 0.001
High school or above	411 (15.0%)	206 (8.9%)	14 (2.5%)	
Marriage				
Other	190 (6.9%)	250 (10.8%)	77 (13.9%)	< 0.001
Married	2556 (93.1%)	2075 (89.2%)	476 (86.1%)	
BMI				
Mean (SD)	23.51 (3.666)	23.88 (4.135)	23.49 (3.778)	0.018
Median [Min, Max]	23.19 [12.56, 62.89]	23.42 [13.57, 60.97]	23.08 [15.39, 48.38]	
BMI_group				
Underweight	123 (4.5%)	134 (5.8%)	45 (8.1%)	< 0.001
Normal weight	1518 (55.3%)	1174 (50.5%)	278 (50.3%)	
Overweight	841 (30.6%)	705 (30.3%)	162 (29.3%)	
Obese	264 (9.7%)	312 (13.4%)	68 (12.3%)	
WC				
Mean (SD)	84.02 (11.55)	84.85 (12.08)	84.89 (12.99)	0.002
Median [Min, Max]	84.00 [19.50, 130.1]	85.00 [21.00, 128.3]	85.80 [21.00, 116.5]	
Smoke				
Never	1530 (55.7%)	1472 (63.3%)	365 (66.0%)	< 0.001
Current	1006 (36.6%)	666 (28.6%)	135 (24.4%)	
Former	210 (7.6%)	187 (8.0%)	53 (9.6%)	
Drink				
Never	1481 (53.9%)	1373 (59.1%)	318 (57.5%)	< 0.001
Current	1108 (40.3%)	757 (32.6%)	145 (26.2%)	
Former	157 (5.7%)	195 (8.4%)	90 (16.3%)	
Sleep				
Poor	531 (19.3%)	946 (40.7%)	327 (59.1%)	< 0.001
Well	2215 (80.7%)	1379 (59.3%)	226 (40.9%)	
SBP				
Mean (SD)	128.0 (19.53)	130.4 (20.87)	133.7 (22.36)	< 0.001
Median [Min, Max]	125.3 [77.00, 233.3]	127.7 [73.00, 223.3]	129.3 [89.70, 196.7]	
DBP				
Mean (SD)	75.57 (11.63)	75.93 (12.00)	75.95 (12.48)	0.541
Median [Min, Max]	74.70 [37.30, 124.3]	75.00 [35.30, 120.0]	75.00 [46.30, 121.0]	
Hypertension				
No	2404 (87.5%)	1592 (68.5%)	325 (58.8%)	< 0.001
Yes	342 (12.5%)	733 (31.5%)	228 (41.2%)	
Diabetes				
No	2691 (98.0%)	2139 (92.0%)	494 (89.3%)	< 0.001
Yes	55 (2.0%)	186 (8.0%)	59 (10.7%)	
Kidney disease				
No	2665 (97.1%)	2189 (94.2%)	509 (92.0%)	< 0.001
Yes	81 (2.9%)	136 (5.8%)	44 (8.0%)	
CRP				
Mean (SD)	2.250 (6.171)	2.626 (6.881)	2.562 (6.686)	< 0.001
Median [Min, Max]	0.9300 [0.03000, 130.4]	1.040 [0.07000, 170.5]	1.050 [0.1700, 78.80]	
FBG				
Mean (SD)	107.3 (29.07)	111.0 (38.14)	113.8 (44.36)	0.006
Median [Min, Max]	101.7 [18.00, 570.8]	102.8 [30.78, 596.2]	102.1 [62.82, 503.8]	
HbAlc				
Mean (SD)	5.187 (0.6649)	5.300 (0.8692)	5.381 (0.9375)	< 0.001
Median [Min, Max]	5.100 [3.500, 12.50]	5.100 [3.500, 12.50]	5.200 [3.700, 12.80]	
TC				
Mean (SD)	192.4 (38.62)	195.4 (38.49)	193.5 (35.17)	0.045
Median [Min, Max]	190.2 [77.71, 627.1]	191.8 [87.37, 460.1]	189.4 [108.6, 335.2]	
TG				
Mean (SD)	133.2 (106.8)	142.3 (130.2)	132.1 (96.47)	0.008
Median [Min, Max]	103.5 [21.24, 1639]	110.6 [2.655, 1905]	108.9 [30.09, 1408]	
HDL				
Mean (SD)	50.67 (15.20)	50.73 (14.97)	51.94 (16.05)	0.238
Median [Min, Max]	49.10 [6.572, 126.0]	49.10 [5.026, 122.6]	50.64 [18.56, 118.7]	
LDL				
Mean (SD)	115.6 (34.84)	117.0 (35.54)	115.6 (32.55)	0.237
Median [Min, Max]	114.0 [2.706, 286.1]	114.8 [0.7732, 277.2]	111.7 [17.01, 264.4]	
Scr				
Mean (SD)	0.7875 (0.1787)	0.7711 (0.1901)	0.7595 (0.1829)	< 0.001
Median [Min, Max]	0.7684 [0.1808, 1.932]	0.7458 [0.1808, 3.379]	0.7345 [0.3164, 1.966]	
BUN				
Mean (SD)	15.49 (4.232)	15.65 (4.414)	15.89 (4.532)	0.325
Median [Min, Max]	14.96 [4.818, 40.03]	15.07 [5.238, 47.53]	15.18 [7.227, 35.21]	
UA				
Mean (SD)	4.505 (1.232)	4.432 (1.231)	4.318 (1.223)	0.001
Median [Min, Max]	4.355 [1.406, 10.49]	4.276 [1.576, 11.01]	4.224 [1.556, 10.57]	
eGFR				
Mean (SD)	94.54 (13.95)	92.97 (14.29)	90.18 (13.71)	< 0.001
Median [Min, Max]	96.85 [31.66, 150.3]	95.74 [18.17, 197.4]	92.23 [29.49, 122.0]	
CKM				
0	215 (7.8%)	133 (5.7%)	30 (5.4%)	< 0.001
1	409 (14.9%)	292 (12.6%)	62 (11.2%)	
2	1473 (53.6%)	1280 (55.1%)	275 (49.7%)	
3	649 (23.6%)	620 (26.7%)	186 (33.6%)	
CVD				
No	2357 (85.8%)	1762 (75.8%)	368 (66.5%)	< 0.001
Yes	389 (14.2%)	563 (24.2%)	185 (33.5%)	

Abbreviations: BMI, Body Mass Index; BUN, Blood Urea Nitrogen; CKM syndrome, Cardiovascular‐Kidney‐Metabolic syndrome; CVD, Cardiovascular disease; DBP, diastolic blood pressure; eGFR, Estimated Glomerular Filtration Rate; FBG, Fasting Blood Glucose; HbA1c, Hemoglobin A1c; HDL, High‐Density Lipoprotein Cholesterol; LDL, Low‐Density Lipoprotein Cholesterol; SBP, Systolic Blood Pressure; Scr, Serum Creatinine; TC, Total Cholesterol; TG, Triglycerides; UA, Uric Acid; WC, Waist Circumference.

### 3.2 Association Between Baseline Frailty and Risk of Cardiovascular Disease

Among participants with CKM syndrome stages 0–3, Kaplan–Meier survival curves demonstrated significant differences in the cumulative risk of CVD across baseline frailty categories (log‐rank test, *p* < 0.001), with a stepwise increase in CVD risk observed with worsening frailty status (Figure S2).

In Cox proportional hazards models, higher baseline frailty was consistently associated with an increased risk of CVD (Table 2). Baseline frailty was evaluated both as a continuous variable using the FI and as a categorical variable based on frailty status. In the unadjusted model (Model I), FI was strongly associated with CVD risk, with each 0.1‐unit increase in FI corresponding to a 38% higher risk of CVD (HR = 1.38, 95% CI: 1.32–1.45). This association remained robust after multivariable adjustment in Model II (HR = 1.33, 95% CI: 1.26–1.40) and Model III (HR = 1.33, 95% CI: 1.26–1.40). Consistent results were observed when frailty was analyzed categorically. Compared with non‐frail participants, those classified as pre‐frail had a significantly higher risk of CVD in Model I (HR = 1.83, 95% CI: 1.61–2.08), which persisted after adjustment in Model II (HR = 1.74, 95% CI: 1.53–1.98) and Model III (HR = 1.72, 95% CI: 1.51–1.97). Participants in the frail group exhibited a further elevation in CVD risk, with HRs of 2.65 (95% CI: 2.22–3.15), 2.32 (95% CI: 1.94–2.78), and 2.29 (95% CI: 1.90–2.76) in Models I, II, and III, respectively. No violation of the proportional hazards assumption was detected based on Schoenfeld residuals.

**TABLE 2:** Relationship between FI and CVD in a population with CKM syndrome stages 0–3.

Outcome	Model I		Model II		Model III	
	HR (95% CI)	*p*	HR (95% CI)	*p*	HR (95% CI)	*p*
FI (per 0.1‐unit)	1.38 (1.32, 1.45)	< 0.001	1.33 (1.26, 1.40)	< 0.001	1.33 (1.26, 1.40)	< 0.001
Frailty status						
Robust	Reference		Reference		Reference	
Pre‐frail	1.83 (1.61, 2.08)	< 0.001	1.74 (1.53, 1.98)	< 0.001	1.72 (1.51, 1.97)	< 0.001
Fail	2.65 (2.22, 3.15)	< 0.001	2.32 (1.94, 2.78)	< 0.001	2.29 (1.90, 2.76)	< 0.001
P for trend	< 0.001		< 0.001		< 0.001	

*Note:* Model I unadjusted. Model II adjusted for age and sex. Model III adjusted for age, sex, Residence, Education, Marriage, BMI, WC, Smoke, Drink, SBP, FBG, HbAlc, TC, TG, HDL, LDL, Scr, BUN, UA, eGFR.

Abbreviations: BMI, body mass index; BUN, blood urea nitrogen; CI, confidence interval; CKM syndrome, Cardiovascular‐Kidney‐Metabolic syndrome; CVD, cardiovascular disease; eGFR, estimated glomerular filtration rate; FBG, fasting blood glucose; FI, frailty index; HbA1c, Hemoglobin A1c; HDL, high‐density lipoprotein cholesterol; HR, Hazard Ratio; LDL, low‐density lipoprotein cholesterol; SBP, systolic blood pressure; Scr, serum creatinine; TC, total cholesterol; TG, triglycerides; UA, uric acid; WC, waist circumference.

To further explore the potential linear or nonlinear relationship between FI and CVD risk, RCS analyses were performed. A significant non‐linear relationship between FI and CVD risk was observed in participants with CKM syndrome stages 0–3 (*P* for non‐linearity < 0.001). As shown in Figure S3, using FI = 0.11 as the reference, CVD risk remained relatively low at lower FI levels, followed by a steep increase with rising FI values; at higher FI levels, the rate of risk increase became less pronounced, although the overall risk continued to rise.

### 3.3 Association Between Cumulative Frailty Index and Risk of Cardiovascular Disease

The cumFI, defined as the sum of FI values across baseline and repeated follow‐up assessments, was used to reflect long‐term cumulative frailty burden. Among participants with CKM syndrome stages 0–3, the cumFI was strongly and independently associated with an increased risk of CVD (Table S5). When modeled as a continuous variable in Cox proportional hazards regression, each 0.1‐unit increase in cumFI was associated with a 15% higher risk of CVD in the unadjusted model (HR = 1.15, 95% CI: 1.14–1.17). This association remained robust after sequential adjustment for age, sex, sociodemographic characteristics, lifestyle factors, and clinical biochemical parameters (fully adjusted model: HR = 1.16, 95% CI: 1.14–1.18). When cumFI was categorized into quartiles, a pronounced and graded increase in CVD risk was observed across higher cumFI categories. Using the lowest quartile (Q1) as the reference group, participants in higher cumFI quartiles exhibited progressively elevated CVD risk. In the fully adjusted model, the risk of CVD increased by 2.36‐fold in Q2 (HR = 2.36, 95% CI: 1.64–3.39), 4.99‐fold in Q3 (HR = 4.99, 95% CI: 3.56–6.98), and 8.46‐fold in Q4 (HR = 8.46, 95% CI: 6.06–11.83), indicating a clear cumulative risk gradient associated with sustained frailty burden over time.

### 3.4 Association Between Frailty Trajectories and Risk of Cardiovascular Disease

**FIGURE 2:**
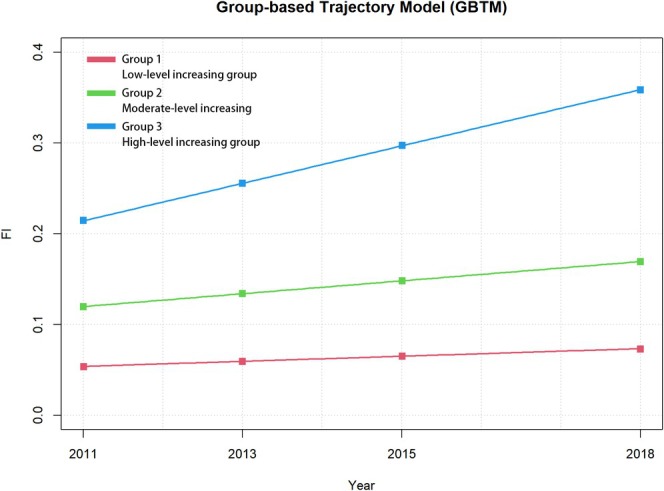
Frailty trajectory groups identified by group‐based trajectory modeling.

Among participants with CKM syndrome stages 0–3, GBTM identified three distinct longitudinal frailty trajectories based on repeated measurements of the FI: a low‐level increasing trajectory, a moderate‐level increasing trajectory, and a high‐level increasing trajectory (Figure 2). Sampled individual frailty trajectories together with the group trajectories are presented in Figure S4. During follow‐up, the numbers (percentages) of incident CVD events were 59 (5.3%), 326 (21.5%), and 229 (38.3%) in the low‐level increasing, moderate‐level increasing, and high‐level increasing groups, respectively. Participants in the low‐level increasing trajectory exhibited the lowest FI values at baseline, with only a mild and gradual increase over time, remaining at an overall low level of frailty throughout follow‐up. The moderate‐level increasing trajectory was characterized by intermediate baseline FI values followed by a steady and sustained increase over time. In contrast, individuals assigned to the high‐level increasing trajectory demonstrated relatively high FI values at baseline and a markedly steeper upward slope during follow‐up, indicating a rapid accumulation of frailty. Overall, these trajectories differed substantially in both initial frailty levels and rates of change, highlighting pronounced heterogeneity in frailty progression within the study population.

Baseline characteristics of participants stratified by frailty trajectory groups are shown in Table S6. During follow‐up, the risk of CVD differed significantly among individuals with different frailty trajectories (Table 3). Using the low‐level increasing trajectory as the reference group, participants in the moderate‐level increasing trajectory exhibited a significantly higher risk of CVD in the fully adjusted model (HR = 4.63, 95% CI: 3.50–6.14). The risk was further amplified among those in the high‐level increasing trajectory (HR = 9.33, 95% CI: 6.89–12.64). When interpreted alongside the trajectory curves, these findings indicate that higher baseline frailty levels combined with more rapid frailty progression over time are associated with progressively greater CVD risk, demonstrating a clear gradient relationship between longitudinal frailty trajectories and cardiovascular outcomes.

**TABLE 3:** Relationship between the frailty developmental trajectory and CVD in a population with CKM syndrome stages 0–3.

Outcome	Model I		Model II		Model III	
	HR (95% CI)	*p*	HR (95% CI)	*p*	HR (95% CI)	*p*
Group						
Low‐level increasing group	Reference		Reference		Reference	
Moderate‐level increasing group	4.46 (3.38, 5.88)	< 0.001	4.49 (3.39, 5.93)	< 0.001	4.63 (3.5, 6.14)	< 0.001
High‐level increasing group	8.84 (6.64, 11.77)	< 0.001	8.97 (6.68, 12.05)	< 0.001	9.33 (6.89, 12.64)	< 0.001

*Note:* Model I unadjusted. Model II adjusted for age and sex. Model III adjusted for age, sex, Residence, Education, Marriage, BMI, WC, Smoke, Drink, SBP, FBG, HbAlc, TC, TG, HDL, LDL, Scr, BUN, UA, eGFR.

Abbreviations: BMI, body mass index; BUN, blood urea nitrogen; CI, confidence interval; CKM syndrome, Cardiovascular‐Kidney‐Metabolic syndrome; CVD, Cardiovascular disease; eGFR, estimated glomerular filtration rate; FBG, fasting blood glucose; HbA1c, hemoglobin A1c; HDL, high‐density lipoprotein cholesterol; HR, Hazard Ratio; LDL, low‐density lipoprotein cholesterol; SBP, systolic blood pressure; Scr, serum creatinine; TC, total cholesterol; TG, triglycerides; UA, uric acid; WC, Waist circumference.

### 3.5 Subgroup and Sensitivity Analyses

Subgroup analyses examining the association between the FI and the risk of CVD among participants with CKM syndrome stages 0–3 are presented in Figure S5. Stratified analyses were conducted according to age, sex, place of residence, marital status, educational attainment, smoking status, alcohol consumption, sleep quality, BMI and CKM syndrome stage. Significant interactions were observed between frailty status and age, marital status, and sleep quality (all *P* for interaction < 0.05), indicating that the association between frailty and CVD risk varied across these subgroups. Although no statistically significant interactions were detected for sex or place of residence, it suggested potential heterogeneity. Across all sensitivity analyses, the results remained materially consistent with those of the primary analyses (Table S7), supporting the stability and reliability of the observed associations.

## 4 Discussion

In this study, focusing on individuals with CKM syndrome stages 0–3, we systematically evaluated the multidimensional associations between frailty and the risk of CVD across three complementary dimensions: baseline status, cumulative burden, and longitudinal trajectories (Figure S6). Our findings demonstrate that both the FI and the cumFI are significantly and positively associated with incident CVD, indicating that frailty represents an independent risk factor for cardiovascular events in the early stages of CKM syndrome, while also highlighting the cumulative burden of frailty on CVD risk over time. Furthermore, different frailty trajectories identified using GBTM exhibited a clear, graded increase in CVD risk, underscoring substantial heterogeneity in frailty progression patterns within this population. Collectively, these results suggest that frailty possesses meaningful prognostic value for cardiovascular risk across CKM syndrome stages 0–3. They further emphasize the importance of comprehensive and longitudinal monitoring of frailty dynamics, which may facilitate the early identification of high‐risk individuals and inform more targeted and effective cardiovascular risk management strategies.

Previous studies have confirmed that frailty is closely related to cardiovascular events and the risk of all‐cause mortality. A prospective study incorporating three cohorts reported that, compared with robust individuals, both pre‐frail and frail participants exhibited a substantially increased risk of CVD [7]. Moreover, the coexistence of frailty and cognitive impairment has been shown to confer markedly higher risks of both all‐cause and cardiovascular mortality [19]. Similarly, a meta‐analysis conducted among individuals with diabetes revealed that frailty and pre‐frailty were significantly associated with elevated risks of all‐cause and cardiovascular mortality [20]. Frailty reflects a global decline in multisystem physiological reserve and impaired homeostatic regulation, which may promote the development of CVD through multiple interconnected biological pathways. Frailty is frequently accompanied by sustained activation of chronic low‐grade inflammation [21], characterized by elevated levels of inflammatory mediators such as CRP, IL‐6, and TNF‐*α*. These proinflammatory factors can directly impair endothelial function [22], accelerate atherosclerotic progression, and increase thrombotic susceptibility [23]. In parallel, frailty is closely linked to disturbances in energy metabolism and insulin resistance [24, 25], leading to reduced glucose uptake and heightened metabolic burden, which may further exacerbate cardiovascular injury through oxidative stress and glucolipotoxic mechanisms. In addition, frailty has been associated with neurotransmitter dysregulation and immune dysfunction [26].

Distinct from prior studies, this study focused on individuals with CKM syndrome stages 0–3, representing a population that has not yet progressed to clinical CVD. According to the AHA, CKM syndrome stage 3 includes individuals with subclinical CVD, which may confer a higher underlying cardiovascular risk and could partly strengthen the observed association between frailty and CVD events. However, this is also consistent with the clinical relevance of the CKM framework, which is intended to identify risk before overt clinical CVD. Our findings demonstrate that higher levels of frailty are associated with a significantly increased risk of future CVD events, suggesting that frailty may serve as an early, identifiable, and clinically meaningful risk marker in the trajectory of CKM syndrome progression. Importantly, these results provide an important supplement to the existing risk assessment system that is based on blood indicators. Using RCS analyses, we identified a clear non‐linear association between FI and CVD risk. At lower FI levels, the increase in CVD risk was relatively modest; however, risk rose more noticeably around FI≈0.11 and then tended to plateau at higher FI levels. Because this value is close to the conventional cut‐off for robust status (FI ≤ 0.10), it may reflect an early transition from relative robustness to increased vulnerability. However, it should be interpreted as a possible risk increase zone rather than a definitive clinical threshold. Compared with a single baseline measurement of the FI, the cumFI provides a more comprehensive representation of an individual's long‐term frailty burden over the follow‐up period. In the present study, cumFI exhibited a strong and graded association with CVD risk, with individuals in the highest quartile experiencing more than an eightfold increase in risk. This finding is highly consistent with the life‐course theory [27], which posits that prolonged exposure to modest but persistent physiological impairment may exert deleterious effects comparable to those of short‐term severe insults. From a clinical perspective, these results suggest that a one‐time frailty assessment may be insufficient to capture an individual's true cardiovascular risk, whereas longitudinal monitoring of frailty evolution may enable more accurate identification of high‐risk individuals. A major innovation of this study lies in the application of frailty trajectory analysis among individuals with CKM syndrome stages 0–3 to systematically evaluate its association with CVD risk. Using GBTM, we identified three distinct frailty trajectories, each demonstrating a clear gradient increase in CVD risk. Notably, individuals following the high‐level rapidly increasing trajectory not only exhibited greater frailty at baseline but also experienced an accelerated progression of frailty over time, translating into a substantially higher risk of CVD compared with other trajectory groups. Such dynamic patterns are difficult to capture using conventional baseline risk stratification approaches. In contrast, trajectory‐based analyses offer a novel and powerful framework for identifying individuals with rapidly deteriorating physiological resilience who may benefit most from early and intensive preventive interventions.

In subgroup analyses, the strength of the association between frailty and CVD risk varied across population strata, with several findings that appear counterintuitive at first glance. The relatively higher risk increase observed in younger populations may reflect the abnormal physiological aging process represented by “early‐onset frailty”. Additionally, married status may represent greater cumulative social role demands and chronic psychosocial stress exposure; once frailty occurs, these factors could amplify its detrimental impact on cardiovascular health. The observation of higher relative risks among participants reporting good sleep quality may likewise reflect a healthy participant effect, whereby the relative contribution of frailty becomes more pronounced in otherwise healthier individuals with lower baseline risk profiles. Collectively, these findings underscore the importance of interpreting frailty‐related cardiovascular risk within a broader context that integrates social‐behavioral factors and baseline health status.

This study carries important clinical and public health implications. From a risk assessment perspective, frailty not only reflects an individual's underlying physiological vulnerability, but its dynamic evolution also captures latent risk that may not be adequately identified by conventional cardiovascular risk factors. As such, frailty assessment may serve as a valuable complementary tool for cardiovascular risk stratification among individuals with CKM syndrome stages 0–3, facilitating earlier identification of high‐risk populations. In clinical practice, frailty assessment could be incorporated into CKM syndrome management as a complementary tool for risk evaluation alongside conventional cardiometabolic indicators. Baseline assessment may support initial risk stratification, whereas repeated assessment over time may help identify worsening vulnerability not fully captured by traditional risk factors. A greater frailty burden may help identify individuals who warrant closer follow‐up and a more comprehensive multidimensional evaluation, including functional status, nutritional condition, and cardiometabolic risk control. From an intervention standpoint, frailty represents a potentially modifiable, multidimensional condition whose development and progression are influenced by insufficient physical activity [28], malnutrition [29], insufficient sleep [30], and chronic low‐grade inflammation [31]. These mechanisms suggest that integrated, multidomain interventions targeting frailty may play a pivotal role in delaying or preventing the transition from CKM syndrome stages 0–3 to overt clinical CVD. Future studies are warranted to determine the reversibility of frailty trajectories and to evaluate whether it can lead to a reduction in cardiovascular risk. Moreover, incorporating frailty trajectories with biomarkers and imaging‐based indicators may enable the development of more precise, dynamic risk prediction models, thereby supporting personalized prevention and management strategies for individuals in the early stages of CKM syndrome. It should also be noted that differences in cardiometabolic profiles, disease burden, and risk factor distributions across populations may influence the generalizability of the AHA‐based CKM syndrome framework. Nevertheless, recent studies have begun to apply this framework in Chinese populations [32, 33, 34], supporting its practical utility in epidemiological research. At the same time, its applicability in Chinese populations still warrants further evaluation.

This study has several notable strengths. First, evidence regarding the association between frailty and CKM syndrome progression remains limited, and to our knowledge, this is the first study to examine the relationship between frailty trajectories and incident CVD among individuals at CKM syndrome stages 0–3. Second, the use of data from a large, nationally representative longitudinal cohort, together with comprehensive adjustment for a wide range of potential confounders, enhances the robustness and generalizability of our findings. Finally, our results underscore the clinical utility of the FI as a simple and scalable tool for identifying individuals at elevated risk of developing CVD in the early stages of CKM syndrome. The consistency of results across multiple sensitivity analyses further supports the reliability of our conclusions. Importantly, this study demonstrates that frailty trajectories can effectively identify individuals at high cardiovascular risk. Several limitations should also be acknowledged. First, consistent with prior studies, CVD outcomes were ascertained based on self‐reported physician diagnoses. Although standardized survey procedures and a built‐in cross‐wave verification procedure for chronic disease reporting were employed, direct validation data for CVD outcomes remain limited, and misclassification bias cannot be completely excluded. In addition, frailer individuals may have more frequent healthcare contact, which could increase the likelihood of CVD reporting, thereby introducing potential detection bias. However, the associations remained materially unchanged after additional adjustment for baseline healthcare utilization. Furthermore, because heart failure was embedded within the self‐reported heart disease category, it could not be evaluated separately. Second, the observational nature of the cohort limits causal inference, and residual confounding may persist despite extensive covariate adjustment. Thirdly, trajectory analyses were based on FI obtained at follow‐up visits, which may not fully capture short‐term fluctuations between assessment intervals. Despite these limitations, the novelty of our study design and the overall robustness of the findings provide important insights and a strong foundation for future research in this area.

## 5 Conclusion

The FI, together with its long‐term accumulation and dynamic progression patterns, was significantly associated with the risk of CKM syndrome progression. Compared with a single baseline assessment, cumulative frailty burden and frailty trajectories demonstrated superior predictive value for risk stratification, indicating that frailty should not be regarded as a static condition but rather as a clinically meaningful dynamic risk phenotype. Notably, trajectory analyses revealed a pronounced gradient in cardiovascular risk across distinct frailty progression patterns, whereby individuals with higher initial frailty levels and more rapid frailty progression over time exhibited substantially elevated risks of cardiovascular events. Collectively, these findings underscore the importance of continuous and dynamic monitoring of frailty in the early stages of CKM syndrome to enable more precise identification of individuals at high cardiovascular risk.

## Author Contributions

H.Y. and R.H. conceived and designed the study. H.Y. performed the data analysis and drafted the manuscript. Z.W. and W.Y. contributed to the interpretation of the data and critically revised the manuscript for important intellectual content. L.M. assisted with data curation and statistical analysis. S.Y. contributed to the acquisition of data and methodological support. All authors reviewed the manuscript, approved the final version, and agreed to be accountable for all aspects of the work.

## Funding

This study was supported by grants from Shenzhen Society of Traditional Chinese Medicine (2024068F), Shenzhen Clinical Research Center for Traditional Chinese Medicine (SZCRC202512), Guangdong Yiyang Healthcare Charity Foundation (JZ2025138), Shenzhen Science and Technology Program (JCYJ20240813152411014), Opening Project of State Key Laboratory of Dampness Syndrome of Chinese Medicine (SZGZZ20240021), Sanming Project of Medicine in Shenzhen (SZZYSM202311004), and Shenzhen Science and Technology Program (JCYJ20240813152342056).

## Ethics Statement

The CHARLS protocol was approved by the Biomedical Ethics Review Committee of Peking University (IRB No. IRB00001052‐11015). All participants provided written informed consent prior to enrollment.

## Supporting information


**Table S1:** The items used to construct the frailty index.
**Table S2:** Criteria for CKM stage classification and definitions of component conditions.
**Table S3:** Framingham risk score.
**Table S4:** GBTM model‐fitting process.
**Table S5:** Relationship between cumFI and CVD in a population with CKM syndrome stages 0–3.
**Table S6:** Baseline characteristics of participants by frailty trajectory groups.
**Table S7:** Sensitivity analysis.
**Figure S1:** Patterns of missing data before and after multiple imputation.
**Figure S2:** Kaplan–Meier curves for incident cardiovascular disease according to baseline frailty status.
**Figure S3:** Restricted cubic spline analysis of the association between the frailty index and cardiovascular disease risk.
**Figure S4:** Sampled individual frailty trajectories together with the group trajectories.
**Figure S5:** Subgroup analyses of the association between frailty index and cardiovascular disease risk among participants with CKM syndrome stages 0–3.
**Figure S6:** Schematic illustration of the study framework.

**Data S1:** Supporting Information.

## Data Availability

The data that support the findings of this study are available in the China Health and Retirement Longitudinal Study at http://charls.pku.edu.cn/en. These data were derived from the following resources available in the public domain: the China Health and Retirement Longitudinal Study, http://charls.pku.edu.cn/en.
