# Supplementary material for: Association of Frailty and Its Trajectories With the Risk of Cardiovascular–Kidney–Metabolic Syndrome Progression: A Longitudinal Cohort Study
Source: Geriatr Gerontol Int. 2026 Jul 2;26(7):e70627. doi: 10.1111/ggi.70627 (PMC13329086; doi:10.1111/ggi.70627)

**Supplementary**

[**Supplementary Table S1. The items used to construct the frailty index** 2](#_Toc225535477)

[**Supplementary Table S2. Criteria for CKM stage classification and definitions of component conditions** 3](#_Toc225535478)

[**Supplementary Table S3. Framingham risk score** 6](#_Toc225535479)

[**Supplementary Figure S1. Patterns of missing data before and after multiple imputation** 8](#_Toc225535480)

[**Supplementary Table S4. GBTM model-fitting process** 9](#_Toc225535481)

[**Supplementary Figure S2.** **Kaplan–Meier curves for incident cardiovascular disease according to baseline frailty status** 10](#_Toc225535482)

[**Supplementary Figure S3. Restricted cubic spline analysis of the association between the frailty index and cardiovascular disease risk** 11](#_Toc225535483)

[**Supplementary Table S5. Relationship between cumFI and CVD in a population with CKM syndrome stages 0–3** 12](#_Toc225535484)

[**Supplementary Figure S4. Sampled individual frailty trajectories together with the group trajectories** 13](#_Toc225535485)

[**Supplementary Table S6. Baseline characteristics of participants by frailty trajectory groups** 14](#_Toc225535486)

[**Supplementary Figure S5. Subgroup analyses of the association between frailty index and cardiovascular disease risk among participants with CKM syndrome stages 0–3** 18](#_Toc225535487)

[**Supplementary Table S7. Sensitivity analysis** 19](#_Toc225535488)

[**Supplementary Figure S6. Schematic illustration of the study framework** 21](#_Toc225535489)

# **Supplementary Table S1. The items used to construct the frailty index**

| **No** | **Description of the items** | **Cut-off value** |
| --- | --- | --- |
| 1 | Self-reported physician diagnosed hypertension | Yes = 1, No = 0 |
| 2 | Self-reported physician diagnosed diabetes | Yes = 1, No = 0 |
| 3 | Self-reported physician diagnosed cancer | Yes = 1, No = 0 |
| 4 | Self-reported physician diagnosed arthritis | Yes = 1, No = 0 |
| 5 | Self-reported physician diagnosed chronic lung disease | Yes = 1, No = 0 |
| 6 | Self-reported physician diagnosed asthma | Yes = 1, No = 0 |
| 7 | Self-reported physician diagnosed any emotional, nervous, or psychiatric problems | Yes = 1, No = 0 |
| 8 | Self-reported physician diagnosed memory-related disease | Yes = 1, No = 0 |
| 9 | Self-reported vision problems | Yes = 1, No = 0 |
| 10 | Self-reported hearing problems | Yes = 1, No = 0 |
| 11 | Difficulty with dressing | Yes = 1, No = 0 |
| 12 | Difficulty with bathing or showering | Yes = 1, No = 0 |
| 13 | Difficulty with eating | Yes = 1, No = 0 |
| 14 | Difficulty with getting in and out of bed | Yes = 1, No = 0 |
| 15 | Difficulty with using the toilet | Yes = 1, No = 0 |
| 16 | Difficulty with managing money | Yes = 1, No = 0 |
| 17 | Difficulty with taking medications | Yes = 1, No = 0 |
| 18 | Difficulty with shopping for groceries | Yes = 1, No = 0 |
| 19 | Difficulty with preparing meals | Yes = 1, No = 0 |
| 20 | Difficulty with doing housework | Yes = 1, No = 0 |
| 21 | Mobility: difficulty with walking 100 yards | Yes = 1, No = 0 |
| 22 | Mobility: difficulty with getting up from a chair after sitting for long periods | Yes = 1, No = 0 |
| 23 | Mobility: difficulty with climbing several flights of stairs without resting | Yes = 1, No = 0 |
| 24 | Mobility: difficulty with lifting or carrying weights over 10 pounds/jins | Yes = 1, No = 0 |
| 25 | Mobility: difficulty with picking up a coin from the table | Yes = 1, No = 0 |
| 26 | Mobility: difficulty with stooping, kneeling, or crouching | Yes = 1, No = 0 |
| 27 | Mobility: difficulty with reaching arms above shoulder level | Yes = 1, No = 0 |
| 28 | Self-reported general health status | Poor or fair = 1, excellent, very good, or good = 0 |
| 29 | Depression: CESD-10 questionnaire | CESD-10 ≤10 =0 >10 =1 |
| 30 | Cognition: (memory test score + orientation test score) **/** 14 | Continuous, ranging from 0 to 1 |

# **Supplementary Table S2. Criteria for CKM stage classification and definitions of component conditions**

**Panel A. Methods for evaluating CKM stages 0-3**

| CKM stages | Threshold for CKM syndrome conditions |
| --- | --- |
| Stages 0 | All criteria are met：   1. BMI< 23 kg/m2 2. Waist circumference <80/90 cm in female/male 3. Fasting blood glucose < 100 mg/dL and HbA1c < 5.7% and without self-reported diagnosis of diabetes, use of insulin, or oral hypoglycemic agents. 4. SBP <130 mm Hg and DBP <80 mm Hg without self-reported diagnosis of hypertension or use of antihypertensive medications. 5. HDL-C <50/40 mg/dL in female/male 6. TG < 150 mg/dL 7. eGFR ≥ 60 ml/min/1.73m2 and without self-reported diagnosis of CKD 8. No Subclinical CVD and clinical CVD |
| Stages 1 | Any of the three criteria is met:   1. Overweight/obesity 2. Abdominal obesity 3. Prediabetes   All criteria are met： （1）SBP <130 mmHg and DBP <80 mmHg without self-reported diagnosis of hypertension or use of antihypertensive medications.  （2）HDL-C <50/40 mg/dL in female/male  （3）TG < 150 mg/dL  （4）eGFR ≥ 60 ml/min/1.73m2 and without self-reported diagnosis of CKD  （5）No Subclinical CVD and clinical CVD |
| Stages 2 | Any of the five criteria is met:   1. Hypertriglyceridemia 2. Hypertension 3. Diabetes 4. metabolic syndrome 5. eGFR：30-60 ml/min/1.73m2 and/or with self-reported diagnosis of CKD   All criteria are met：   1. No Subclinical CVD and clinical CVD |
| Stages 3 | Any of the two criteria is met:   1. eGFR<30 ml/min/1.73m2 2. Subclinical CVD   Any of the eight criteria is met:   1. Overweight/obesity 2. Abdominal obesity 3. Prediabetes 4. Hypertriglyceridemia 5. Hypertension 6. diabetes 7. metabolic syndrome 8. eGFR：30-60 ml/min/1.73m2 and/or with self-reported diagnosis of CKD   The criterion is met：   1. No clinical CVD |

Abbreviations: BMI, body mass index; CKD, chronic kidney disease; CKM syndrome, cardiovascular-kidney-metabolic syndrome; CVD, cardiovascular disease; SBP, systolic blood pressure; DBP, diastolic blood pressure; eGFR, estimated glomerular filtration rate; HDL-C, high-density lipoprotein cholesterol; HbA1c, Hemoglobin A1c; TG, Triglycerides.

**Panel B. Specific definitions of various diseases**

| Overweight/obesity | BMI ≥23 kg/m2 |
| --- | --- |
| Abdominal obesity | Waist circumference ≥80/90 cm in female/male |
| Prediabetes | Fasting blood glucose ≥ 100-124 mg/dL or HbA1c ≥ 5.7%-6.4% and  without self-reported diagnosis of diabetes, use of insulin, or oral  hypoglycemic agents |
| Diabetes | Fasting blood glucose ≥ 125 mg/dL or HbA1c ≥ 6.5% or self-reported diagnosis of diabetes, use of insulin, or oral hypoglycemic agents |
| Hypertension | SBP ≥130 mm Hg or DBP ≥80 mm Hg or self-reported diagnosis of hypertension or use of antihypertensive medications |
| chronic kidney disease | Any of the two criteria is met：   1. Self-reported history of chronic kidney disease 2. eGFR < 60 ml/min/1.73m2 |
| Hypertriglyceridemia | Triglycerides ≥ 135 mg/dL |
| metabolic syndrome | Meet any three or more of the five  ① Waist circumference ≥ 80/90 cm in female/male.  ② HDL-C ≥50/40 mg/dL in female/male.  ③ Triglycerides ≥150 mg/dL.  ④ Elevated blood pressure (SBP ≥130 mm Hg or DBP ≥80 mm Hg and/or use of antihypertensive medications)  ⑤ Fasting blood glucose ≥100 mg/dL |
| Clinical CVD | Any of the two criteria is met： ① Participants were asked "Have you been told by a doctor that you have been diagnosed with a heart disease, including angina, heart attack, congestive heart failure, and other heart problems?" ②"Have you been told by a doctor that you have been diagnosed with a stroke?" |
| Subclinical CVD | Any of the two criteria is met：   1. Very high-risk CKD in KDIGO classification: eGFR < 30 ml/min/1.73m2. 2. Framingham risk score ≥21.5/21.6 in female/male |

Abbreviations: BMI, body mass index; CKD, chronic kidney disease; CVD, cardiovascular disease; SBP, systolic blood pressure; DBP, diastolic blood pressure; eGFR, estimated glomerular filtration rate; HDL-C, high-density lipoprotein cholesterol; HbA1c, Hemoglobin A1c; KDIGO, The Kidney Disease: Improving Global Outcomes.

# **Supplementary Table S3. Framingham risk score**

**Panel A. Framingham risk score for Men**

| **Points** | **Age, years** | **HDL-C** | **TC** | **SBP Not Treated** | **SBP Treated** | **Smoker** | **Diabetic** |
| --- | --- | --- | --- | --- | --- | --- | --- |
| -2 |  | 60+ |  | <120 |  |  |  |
| -1 |  | 50–59 |  |  |  |  |  |
| 0 | 30–34 | 45–49 | <160 | 120–129 | <120 | No | No |
| 1 |  | 35–44 | 160–199 | 130–139 |  |  |  |
| 2 | 35–39 | <35 | 200–239 | 140–159 | 120–129 |  |  |
| 3 |  |  | 240–279 | 160+ | 130–139 |  | Yes |
| 4 |  |  | 280+ |  | 140–159 | Yes |  |
| 5 | 40–44 |  |  |  | 160+ |  |  |
| 6 | 45–49 |  |  |  |  |  |  |
| 7 |  |  |  |  |  |  |  |
| 8 | 50–54 |  |  |  |  |  |  |
| 9 |  |  |  |  |  |  |  |
| 10 | 55–59 |  |  |  |  |  |  |
| 11 | 60–64 |  |  |  |  |  |  |
| 12 | 65–69 |  |  |  |  |  |  |
| 13 |  |  |  |  |  |  |  |
| 14 | 70–74 |  |  |  |  |  |  |
| 15 | 75+ |  |  |  |  |  |  |

Abbreviations: SBP, systolic blood pressure; HDL-C, high-density lipoprotein cholesterol; TC, Total Cholesterol.

**Panel B. Framingham risk score for Women**

| **Points** | **Age, years** | **HDL** | **TC** | **SBP Not Treated** | **SBP Treated** | **Smoker** | **Diabetic** |
| --- | --- | --- | --- | --- | --- | --- | --- |
| -3 |  |  |  | <120 |  |  |  |
| -2 |  | 60+ |  |  |  |  |  |
| -1 |  | 50–59 |  |  | <120 |  |  |
| 0 | 30–34 | 45–49 | <160 | 120–129 |  | No | No |
| 1 |  | 35–44 | 160–199 | 130–139 |  |  |  |
| 2 | 35–39 | <35 |  | 140–149 | 120–129 |  |  |
| 3 |  |  | 200–239 |  | 130–139 | Yes |  |
| 4 | 40–44 |  | 240–279 | 150–159 |  |  | Yes |
| 5 | 45–49 |  | 280+ | 160+ | 140–149 |  |  |
| 6 |  |  |  |  | 150–159 |  |  |
| 7 | 50–54 |  |  |  | 160+ |  |  |
| 8 | 55–59 |  |  |  |  |  |  |
| 9 | 60–64 |  |  |  |  |  |  |
| 10 | 65–69 |  |  |  |  |  |  |
| 11 | 70–74 |  |  |  |  |  |  |
| 12 | 75+ |  |  |  |  |  |  |

Abbreviations: SBP, systolic blood pressure; HDL-C, high-density lipoprotein cholesterol; TC, Total Cholesterol.

# **Supplementary Figure S1. Patterns of missing data before and after multiple imputation**


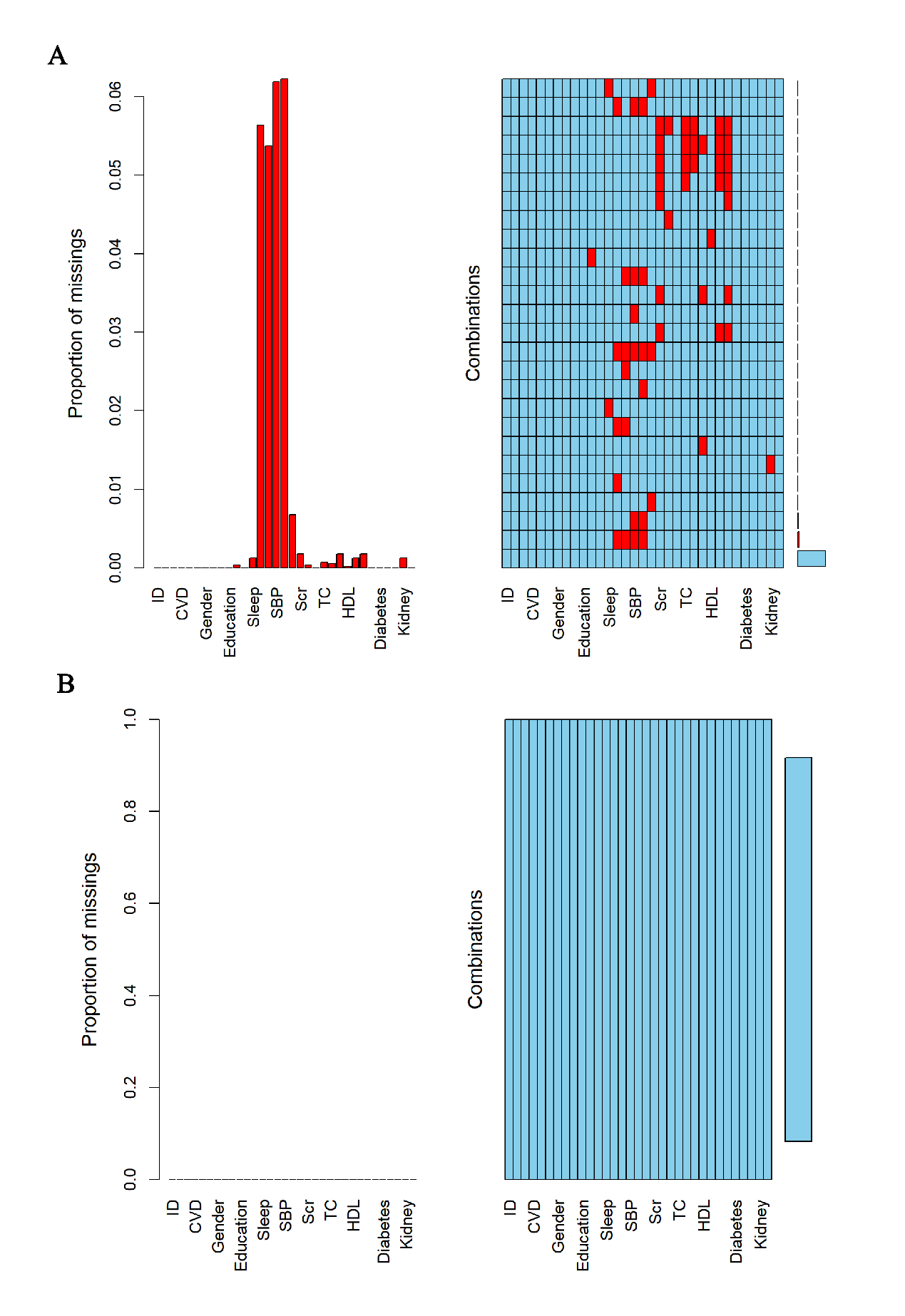


**Supplementary Table S4. GBTM model-fitting process**

| Number of classes | AIC | BIC | AvePP | %class1 | %class2 | %class3 | %class4 | %class5 |
| --- | --- | --- | --- | --- | --- | --- | --- | --- |
| 1 | -21701.58 | -21680.18 | 1.00 | 100.00 |  |  |  |  |
| 2 | -29475.06 | -29422.79 | 0.96/0.96 | 63.95 | 36.05 |  |  |  |
| 3 | -32110.72 | -32028.58 | 0.93/0.92/0.95 | 34.50 | 47.00 | 18.50 |  |  |
| 4 | -33040.01 | -32928.01 | 0.91/0.88/0.94/0.89 | 15.66 | 36.08 | 14.39 | 33.88 |  |
| 5 | -33338.28 | -33181.47 | 0.89/0.85/0.84/0.90/0.84 | 12.38 | 28.53 | 32.77 | 7.21 | 19.12 |

Abbreviations: AIC, Akaike Information Criterion; BIC, Bayesian Information Criterion; AvePP, average posterior probability of assignment

# **Supplementary Figure S2.** **Kaplan–Meier curves for incident cardiovascular disease according to baseline frailty status**





# **Supplementary Figure S3. Restricted cubic spline analysis of the association between the frailty index and cardiovascular disease risk**





**Supplementary Table S5. Relationship between cumFI and CVD in a population with CKM syndrome stages 0–3**

| Outcome | Model Ⅰ | |  | Model Ⅱ | |  | Model Ⅲ | |
| --- | --- | --- | --- | --- | --- | --- | --- | --- |
|  | HR (95%CI) | P-value |  | HR (95%CI) | P-value |  | HR (95%CI) | P-value |
| cumFI (per 0.1-unit) | 1.15 (1.14, 1.17) | <0.001 |  | 1.16 (1.14, 1.18) | <0.001 |  | 1.16 (1.14, 1.18) | <0.001 |
| cumFI (quartile) |  |  |  |  |  |  |  |  |
| Q1 | Reference |  |  | Reference |  |  | Reference |  |
| Q2 | 2.28 (1.59, 3.28) | <0.001 |  | 2.29 (1.59, 3.28) | <0.001 |  | 2.36 (1.64, 3.39) | <0.001 |
| Q3 | 4.77 (3.43, 6.65) | <0.001 |  | 4.79 (3.44, 6.69) | <0.001 |  | 4.99 (3.56, 6.98) | <0.001 |
| Q4 | 8.17 (5.93, 11.25) | <0.001 |  | 8.23 (5.93, 11.41) | <0.001 |  | 8.46 (6.06, 11.83) | <0.001 |
| P for trend | <0.001 |  |  | < 0.001 |  |  | <0.001 |  |

Model Ⅰ unadjusted.

Model Ⅱ adjusted for age and Sex.

Model Ⅲ adjusted for age, Sex, Residence, Education, Marriage, BMI, WC, Smoke, Drink, SBP, FBG, HbAlc, TC, TG, HDL, LDL, Scr, BUN, UA, eGFR

Abbreviations: cumFI, cumulative Frailty Index; CVD, Cardiovascular disease; CKM syndrome, Cardiovascular-Kidney-Metabolic syndrome; HR, Hazard Ratio; CI, confidence interval; BMI, Body Mass Index; WC, Waist Circumference; SBP, Systolic Blood Pressure; FBG, Fasting Blood Glucose; HbA1c, Hemoglobin A1c; TC, Total Cholesterol; TG, Triglycerides; HDL, High-Density Lipoprotein Cholesterol; LDL, Low-Density Lipoprotein Cholesterol; Scr, Serum Creatinine; BUN, Blood Urea Nitrogen; UA, Uric Acid; eGFR, Estimated Glomerular Filtration Rate.

# **Supplementary Figure S4. Sampled individual frailty trajectories together with the group trajectories**


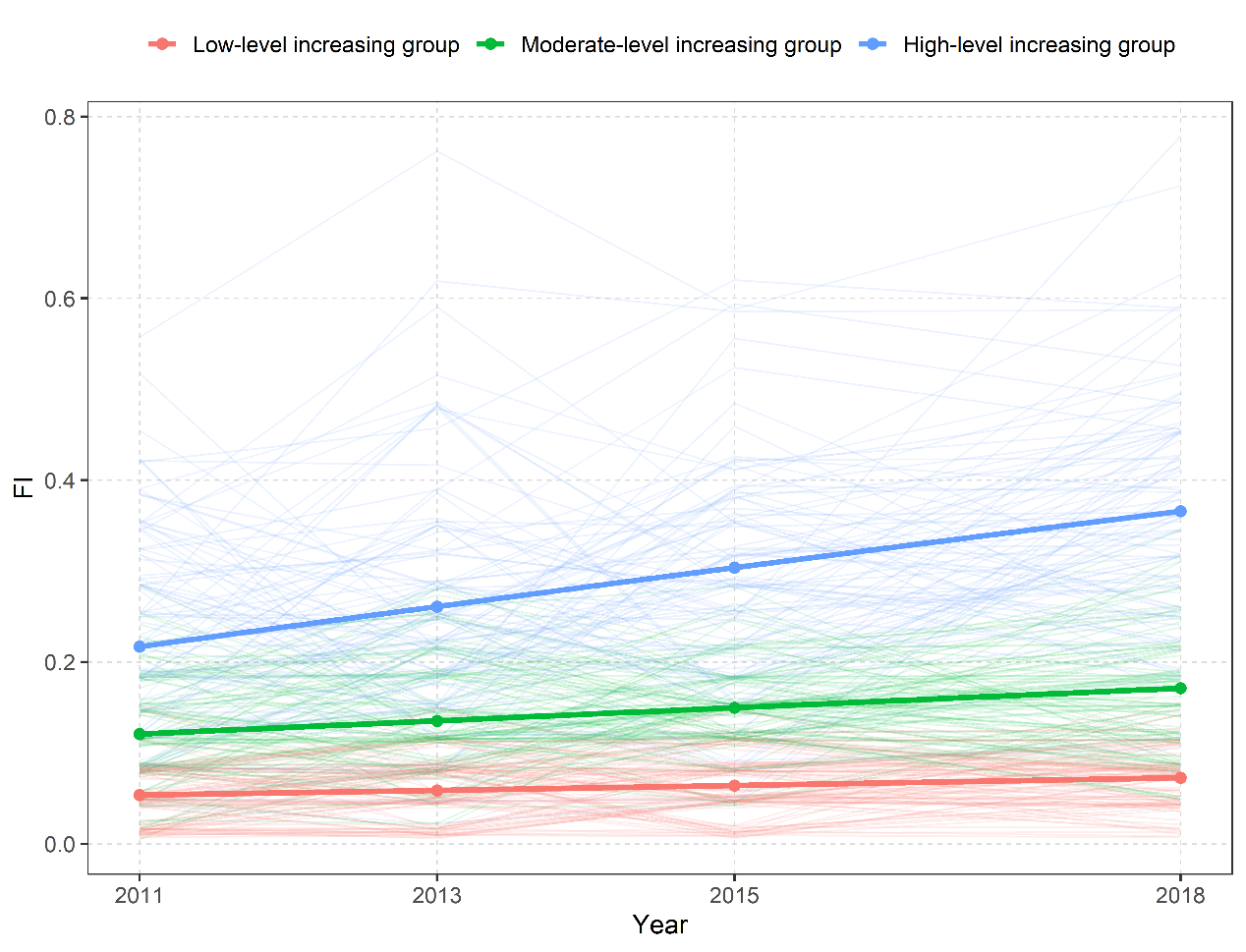


# **Supplementary Table S6. Baseline characteristics of participants by frailty trajectory groups**

|  | Group 1 (N= 1115) | Group 2 (N= 1519) | Group 3 (N= 598) | P-value |
| --- | --- | --- | --- | --- |
| **Age** |  |  |  |  |
| Mean (SD) | 54.99 (7.472) | 56.64 (7.661) | 59.77 (7.665) | <0.001 |
| Median [Min, Max] | 54.00 [45.00, 101.0] | 56.00 [45.00, 88.00] | 60.00 [45.00, 83.00] |  |
| **Sex** |  |  |  |  |
| female | 425 (38.1%) | 768 (50.6%) | 366 (61.2%) | <0.001 |
| male | 690 (61.9%) | 751 (49.4%) | 232 (38.8%) |  |
| **Residence** |  |  |  |  |
| Urban | 475 (42.6%) | 548 (36.1%) | 177 (29.6%) | <0.001 |
| Rural | 640 (57.4%) | 971 (63.9%) | 421 (70.4%) |  |
| **Education** |  |  |  |  |
| Middle school or below | 884 (79.3%) | 1333 (87.8%) | 567 (94.8%) | <0.001 |
| high school or above | 231 (20.7%) | 186 (12.2%) | 31 (5.2%) |  |
| **Marriage** |  |  |  |  |
| Other | 56 (5.0%) | 106 (7.0%) | 62 (10.4%) | <0.001 |
| Married | 1059 (95.0%) | 1413 (93.0%) | 536 (89.6%) |  |
| **BMI** |  |  |  |  |
| Mean (SD) | 23.71 (3.802) | 23.99 (3.849) | 24.09 (4.153) | 0.124 |
| Median [Min, Max] | 23.32 [16.12, 62.89] | 23.53 [12.97, 51.53] | 23.57 [14.51, 48.38] |  |
| **Smoke** |  |  |  |  |
| Never | 602 (54.0%) | 911 (60.0%) | 390 (65.2%) | <0.001 |
| Current | 418 (37.5%) | 480 (31.6%) | 145 (24.2%) |  |
| Former | 95 (8.5%) | 128 (8.4%) | 63 (10.5%) |  |
| **Drink** |  |  |  |  |
| Never | 561 (50.3%) | 850 (56.0%) | 379 (63.4%) | <0.001 |
| Current | 498 (44.7%) | 551 (36.3%) | 152 (25.4%) |  |
| Former | 56 (5.0%) | 118 (7.8%) | 67 (11.2%) |  |
| **Sleep** |  |  |  |  |
| poor | 212 (19.0%) | 472 (31.1%) | 295 (49.3%) | <0.001 |
| well | 903 (81.0%) | 1047 (68.9%) | 303 (50.7%) |  |
| **SBP** |  |  |  |  |
| Mean (SD) | 125.4 (17.32) | 128.9 (19.97) | 131.6 (20.97) | <0.001 |
| Median [Min, Max] | 123.3 [83.70, 196.3] | 126.3 [89.70, 216.7] | 128.3 [89.70, 196.7] |  |
| **DBP** |  |  |  |  |
| Mean (SD) | 75.08 (11.22) | 75.89 (11.90) | 76.15 (11.53) | 0.168 |
| Median [Min, Max] | 74.30 [44.00, 120.3] | 75.00 [37.30, 123.7] | 75.00 [45.00, 121.0] |  |
| **Hypertension** |  |  |  |  |
| no | 1017 (91.2%) | 1097 (72.2%) | 369 (61.7%) | <0.001 |
| yes | 98 (8.8%) | 422 (27.8%) | 229 (38.3%) |  |
| **Diabetes** |  |  |  |  |
| no | 1097 (98.4%) | 1414 (93.1%) | 525 (87.8%) | <0.001 |
| yes | 18 (1.6%) | 105 (6.9%) | 73 (12.2%) |  |
| **Kidney Disease** |  |  |  |  |
| no | 1083 (97.1%) | 1444 (95.1%) | 554 (92.6%) | <0.001 |
| yes | 32 (2.9%) | 75 (4.9%) | 44 (7.4%) |  |
| **CRP** |  |  |  |  |
| Mean (SD) | 1.926 (4.745) | 2.458 (6.964) | 3.070 (8.171) | <0.001 |
| Median [Min, Max] | 0.8400 [0.06000, 66.40] | 0.9800 [0.09000, 170.5] | 1.160 [0.1600, 110.2] |  |
| **FBG** |  |  |  |  |
| Mean (SD) | 105.7 (27.96) | 110.2 (35.49) | 112.4 (42.80) | 0.044 |
| Median [Min, Max] | 101.7 [36.72, 570.8] | 102.4 [36.54, 504.4] | 101.7 [30.78, 503.8] |  |
| **HbAlc** |  |  |  |  |
| Mean (SD) | 5.138 (0.6133) | 5.270 (0.8302) | 5.393 (0.9514) | <0.001 |
| Median [Min, Max] | 5.100 [3.500, 12.50] | 5.100 [3.500, 11.70] | 5.200 [4.000, 12.80] |  |
| **TC** |  |  |  |  |
| Mean (SD) | 191.1 (37.12) | 194.2 (36.92) | 194.0 (38.76) | 0.081 |
| Median [Min, Max] | 187.9 [78.09, 358.0] | 191.8 [89.30, 460.1] | 189.4 [105.5, 344.8] |  |
| **TG** |  |  |  |  |
| Mean (SD) | 131.1 (93.49) | 142.7 (133.0) | 140.5 (108.9) | 0.031 |
| Median [Min, Max] | 104.4 [25.67, 1049] | 109.7 [2.655, 1905] | 114.2 [38.06, 1408] |  |
| **HDL** |  |  |  |  |
| Mean (SD) | 49.94 (14.44) | 50.53 (15.68) | 50.42 (15.34) | 0.629 |
| Median [Min, Max] | 48.33 [14.69, 102.8] | 48.71 [9.278, 123.3] | 49.10 [5.026, 118.7] |  |
| **LDL** |  |  |  |  |
| Mean (SD) | 115.3 (34.27) | 115.9 (35.11) | 116.4 (35.88) | 0.956 |
| Median [Min, Max] | 114.0 [14.30, 286.1] | 114.4 [2.706, 277.2] | 112.7 [0.7732, 264.4] |  |
| **Scr** |  |  |  |  |
| Mean (SD) | 0.7906 (0.1711) | 0.7772 (0.1824) | 0.7824 (0.2177) | 0.024 |
| Median [Min, Max] | 0.7797 [0.3390, 1.401] | 0.7571 [0.2486, 1.819] | 0.7571 [0.3164, 3.379] |  |
| **BUN** |  |  |  |  |
| Mean (SD) | 15.29 (4.098) | 15.65 (4.447) | 15.67 (4.423) | 0.243 |
| Median [Min, Max] | 14.85 [4.818, 36.64] | 15.04 [6.386, 40.03] | 14.90 [6.106, 47.53] |  |
| **UA** |  |  |  |  |
| Mean (SD) | 4.537 (1.246) | 4.452 (1.218) | 4.387 (1.239) | 0.023 |
| Median [Min, Max] | 4.368 [1.482, 10.17] | 4.267 [1.576, 9.860] | 4.250 [1.556, 10.57] |  |
| **eGFR** |  |  |  |  |
| Mean (SD) | 96.10 (13.11) | 94.34 (13.90) | 90.37 (14.53) | <0.001 |
| Median [Min, Max] | 98.37 [46.33, 149.3] | 96.98 [31.66, 146.3] | 93.25 [18.17, 121.8] |  |
| **CKM** |  |  |  |  |
| 0 | 97 (8.7%) | 97 (6.4%) | 35 (5.9%) | <0.001 |
| 1 | 188 (16.9%) | 200 (13.2%) | 65 (10.9%) |  |
| 2 | 590 (52.9%) | 843 (55.5%) | 316 (52.8%) |  |
| 3 | 240 (21.5%) | 379 (25.0%) | 182 (30.4%) |  |
| **CVD** |  |  |  |  |
| no | 1056 (94.7%) | 1193 (78.5%) | 369 (61.7%) | <0.001 |
| yes | 59 (5.3%) | 326 (21.5%) | 229 (38.3%) |  |

Abbreviations: CVD, Cardiovascular disease; CKM syndrome, Cardiovascular-Kidney-Metabolic syndrome; BMI, Body Mass Index; WC, Waist Circumference; SBP, Systolic Blood Pressure; DBP, diastolic blood pressure; FBG, Fasting Blood Glucose; HbA1c, Hemoglobin A1c; TC, Total Cholesterol; TG, Triglycerides; HDL, High-Density Lipoprotein Cholesterol; LDL, Low-Density Lipoprotein Cholesterol; Scr, Serum Creatinine; BUN, Blood Urea Nitrogen; UA, Uric Acid; eGFR, Estimated Glomerular Filtration Rate.

# **Supplementary Figure S5. Subgroup analyses of the association between frailty index and cardiovascular disease risk among participants with CKM syndrome stages 0–3**


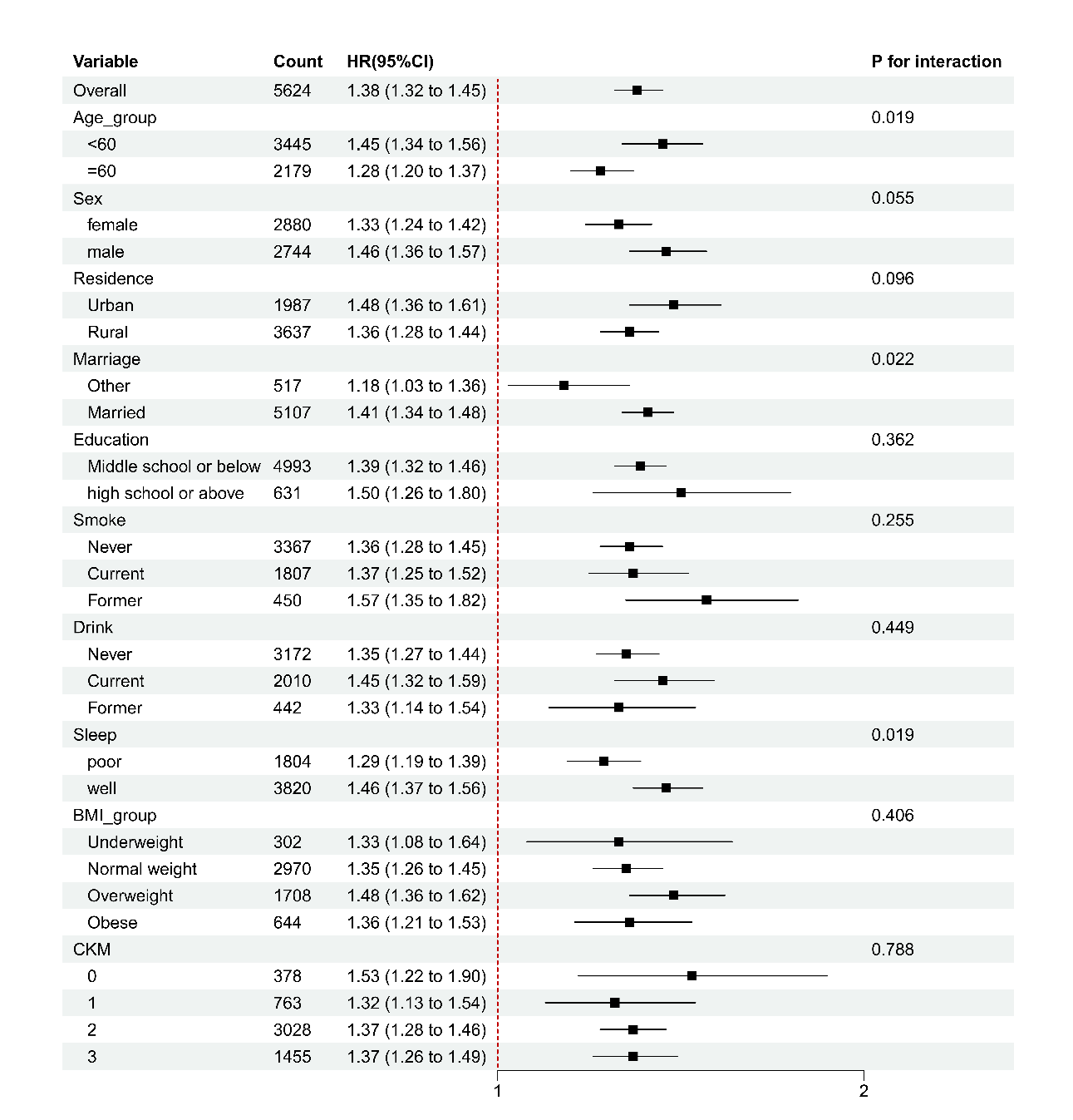


# **Supplementary Table S7. Sensitivity analysis**

**Panel A. Sensitivity analyses using alternative sample restrictions and analytical approaches**

| Outcome | Exclude participants with outlier values in baseline FI | |  | Association between FI and CVD assessed by logistic regression | |  | Exclude participants who developed CVD at the second follow-up | |
| --- | --- | --- | --- | --- | --- | --- | --- | --- |
|  | HR (95%CI) ^a^ | P-value ^a^ |  | OR (95%CI) ^a^ | P-value ^a^ |  | HR (95%CI) ^a^ | P-value ^a^ |
| FI (per 0.1-unit) | 1.52(1.41, 1.65) | <0.001 |  | 1.44(1.35, 1.55) | <0.001 |  | 1.34 (1.26, 1.42) | <0.001 |
| Frailty status |  |  |  |  |  |  |  |  |
| Robust | Reference |  |  | Reference |  |  | Reference |  |
| Pre-fail | 1.72 (1.50, 1.96) | <0.001 |  | 1.81 (1.56, 2.10) | <0.001 |  | 1.71(1.48, 1.98) | <0.001 |
| Fail | 2.14 (1.71, 2.68) | <0.001 |  | 2.59 (2.07, 3.23) | <0.001 |  | 2.35(1.92, 2.89) | <0.001 |

^a^ HR, OR and P-value were adjusted for age, Sex, Residence, Education, Marriage, BMI, WC, Smoke, Drink, SBP, FBG, HbAlc, TC, TG, HDL, LDL, Scr, BUN, UA, eGFR.

Abbreviations: FI, Frailty Index; CVD, Cardiovascular disease; HR, Hazard Ratio; CI, confidence interval; OR, Odds Ratio**;** BMI, Body Mass Index; WC, Waist Circumference; SBP, Systolic Blood Pressure; FBG, Fasting Blood Glucose; HbA1c, Hemoglobin A1c; TC, Total Cholesterol; TG, Triglycerides; HDL, High-Density Lipoprotein Cholesterol; LDL, Low-Density Lipoprotein Cholesterol; Scr, Serum Creatinine; BUN, Blood Urea Nitrogen; UA, Uric Acid; eGFR, Estimated Glomerular Filtration Rate.

**Panel B. Sensitivity analyses using alternative frailty definitions and adjustment strategies**

| Outcome | modified FI excluding hypertension and diabetes | |  | Additional adjustment for healthcare utilization | |  | Additional adjustment for Framingham risk score | |
| --- | --- | --- | --- | --- | --- | --- | --- | --- |
|  | HR (95%CI) ^a^ | P-value ^a^ |  | HR (95%CI) ^a^ | P-value ^a^ |  | HR (95%CI) ^a^ | P-value ^a^ |
| FI (per 0.1-unit) | 1.29(1.23, 1.35) | <0.001 |  | 1.32(1.25, 1.39) | <0.001 |  | 1.33 (1.26, 1.40) | <0.001 |
| Frailty status |  |  |  |  |  |  |  |  |
| Robust | Reference |  |  | Reference |  |  | Reference |  |
| Pre-fail | 1.72 (1.49, 1.98) | <0.001 |  | 1.70 (1.49, 1.94) | <0.001 |  | 1.73(1.51, 1.97) | <0.001 |
| Fail | 2.45 (2.02, 2.97) | <0.001 |  | 2.22 (1.83, 2.68) | <0.001 |  | 2.29(1.90, 2.76) | <0.001 |

^a^ HR and P-value were adjusted for age, Sex, Residence, Education, Marriage, BMI, WC, Smoke, Drink, SBP, FBG, HbAlc, TC, TG, HDL, LDL, Scr, BUN, UA, eGFR.

Abbreviations: FI, Frailty Index; CVD, Cardiovascular disease; HR, Hazard Ratio; CI, confidence interval; BMI, Body Mass Index; WC, Waist Circumference; SBP, Systolic Blood Pressure; FBG, Fasting Blood Glucose; HbA1c, Hemoglobin A1c; TC, Total Cholesterol; TG, Triglycerides; HDL, High-Density Lipoprotein Cholesterol; LDL, Low-Density Lipoprotein Cholesterol; Scr, Serum Creatinine; BUN, Blood Urea Nitrogen; UA, Uric Acid; eGFR, Estimated Glomerular Filtration Rate.

# **Supplementary Figure S6. Schematic illustration of the study framework**


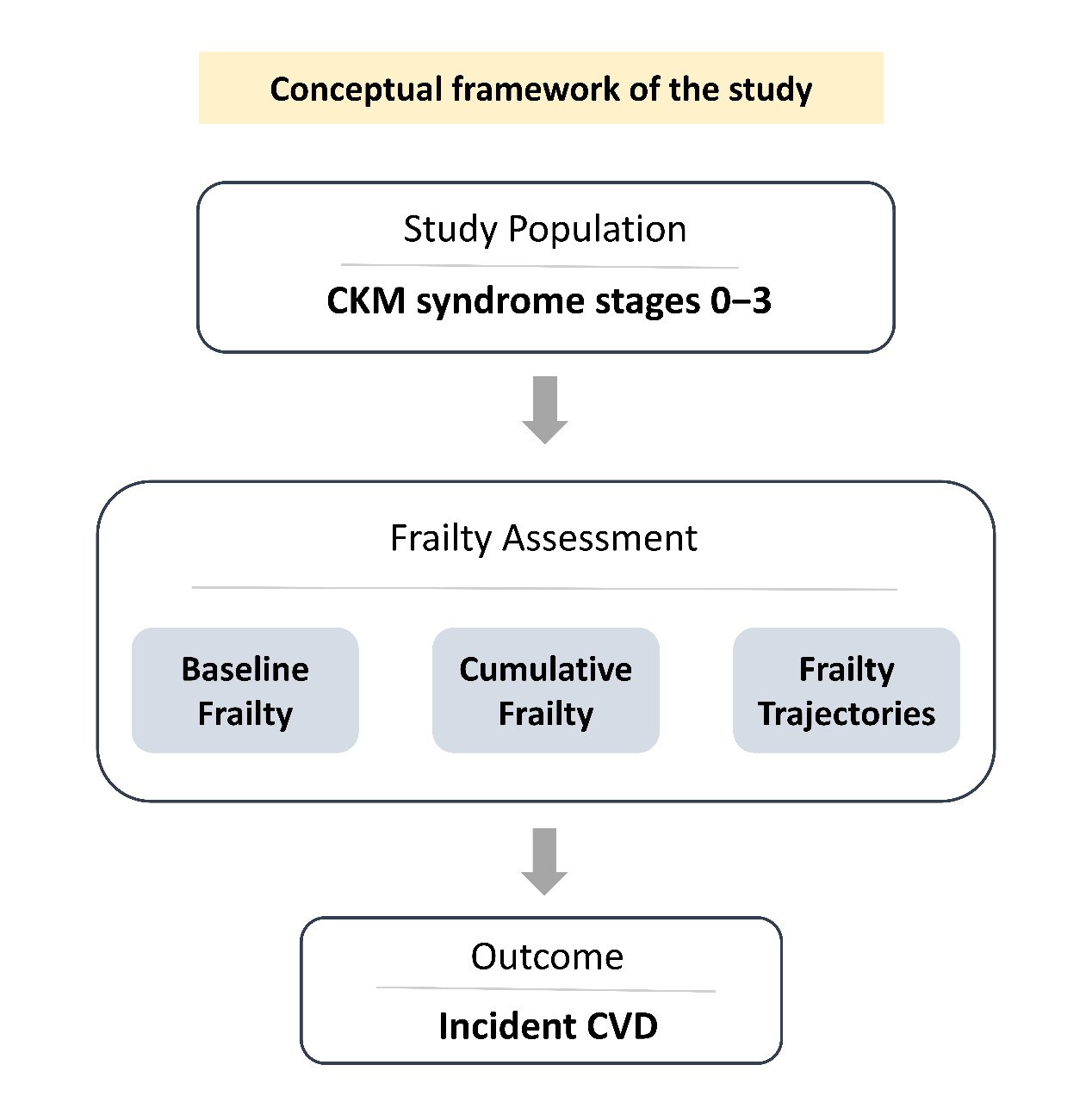

Supplement: Supplementary file 1 — Table S1: The items used to construct the frailty index. Table S2: Criteria for CKM stage classification and definitions of component conditions. Table S3: Framingham risk score. Table S4: GBTM model‐fitting process. Table S5: Relationship between cumFI and CVD in a population with CKM syndrome stages 0–3. Table S6: Baseline characteristics of participants by frailty trajectory groups. Table S7: Sensitivity analysis. Figure S1: Patterns of missing data before and after multiple imputation. Figure S2: Kaplan–Meier curves for incident cardiovascular disease according to baseline frailty status. Figure S3: Restricted cubic spline analysis of the association between the frailty index and cardiovascular disease risk. Figure S4: Sampled individual frailty trajectories together with the group trajectories. Figure S5: Subgroup analyses of the association between frailty index and cardiovascular disease risk among participants with CKM syndrome stages 0–3. Figure S6: Schematic illustration of the study framework. [file GGI-26-0-s001.docx]
