# Supplementary figures and images for: Association of Frailty and Its Trajectories With the Risk of Cardiovascular–Kidney–Metabolic Syndrome Progression: A Longitudinal Cohort Study
Source: Geriatr Gerontol Int. 2026 Jul 2;26(7):e70627. doi: 10.1111/ggi.70627 (PMC13329086; doi:10.1111/ggi.70627)

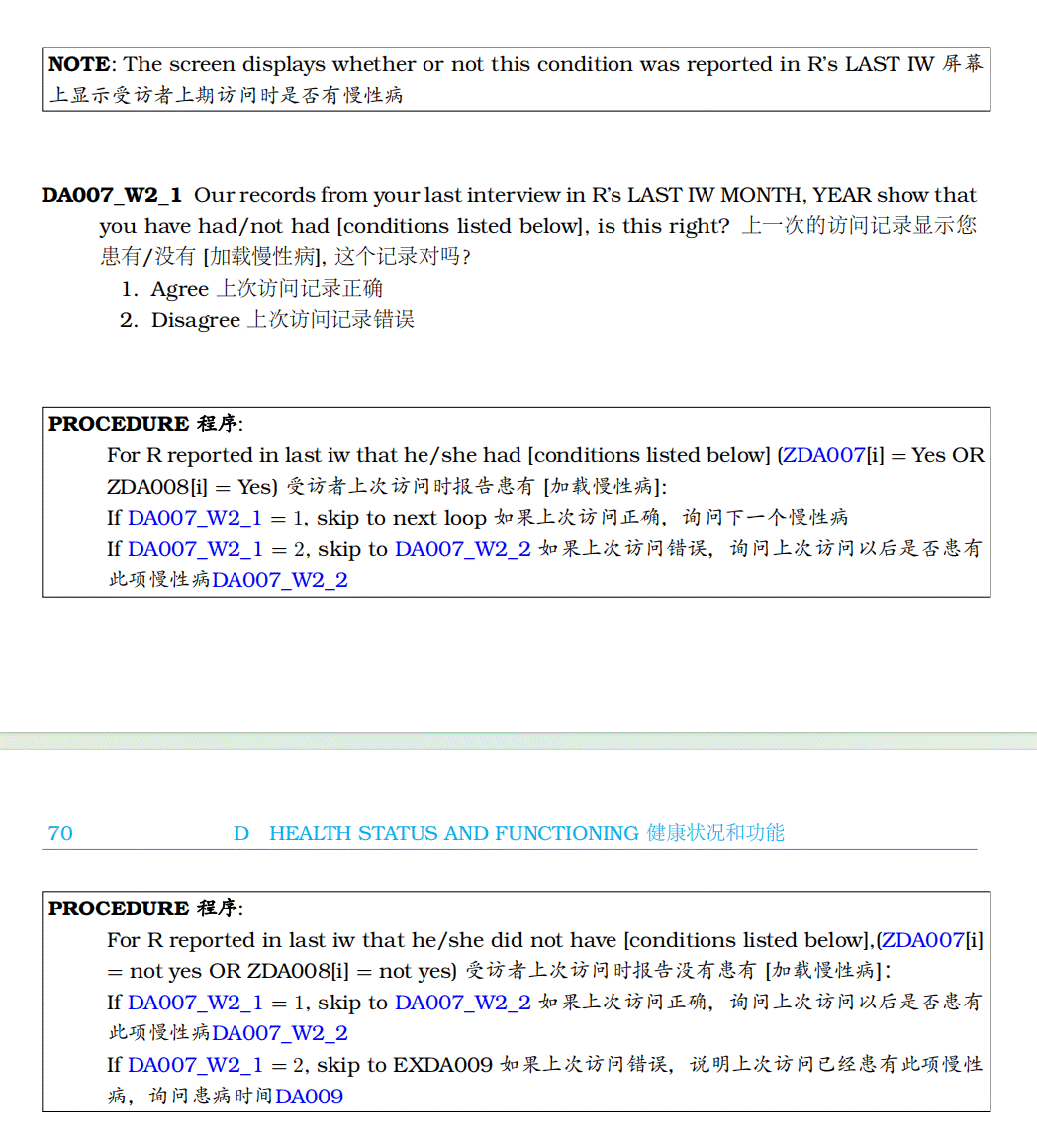

Supplement: Supplementary file 2 — Data S1: Supporting Information. [file GGI-26-0-s002.gif]
